# Impact of a pharmacist driven antimicrobial stewardship program on inpatient antibiotic consumption in a Chinese Tertiary Hospital: a 5-year retrospective study

**DOI:** 10.3389/fmed.2025.1583134

**Published:** 2025-06-25

**Authors:** Can Qian, Ting Yuan, Fuyong Zhang, Shiyuan Gou, Chunmei Jiang, Xuebing Chen, Ya Huang, Xiaolin Zhao, You Du, Chenglong Li, Yangyun Han, Huaiyu Su

**Affiliations:** ^1^Department of Pharmacy, Deyang People’s Hospital, Deyang, China; ^2^Department of Medical Affairs, Deyang People’s Hospital, Deyang, China; ^3^Department of Operation Management, Deyang People’s Hospital, Deyang, China; ^4^Department of Infectious Diseases, Deyang People’s Hospital, Deyang, China; ^5^Office of the Hospital President, Deyang People’s Hospital, Deyang, China

**Keywords:** antimicrobial stewardship, hospital pharmacy, antibiotic consumption, antibiotic use density, rational drug use

## Abstract

**Introduction:**

Antimicrobial Stewardship programs are crucial for reducing overall antibiotic consumption, but in practice, there are often issues with unclear responsibilities and ambiguous tasks. Pharmacists play a critical role in AMS due to their combined management functions and professional expertise.

**Objective:**

To investigate the impact of a Pharmacist-Driven Antimicrobial Stewardship program on the consumption of antibiotics in a hospital.

**Methods:**

Under the support of a hospital top-level design, we implemented a Pharmacist-Driven Antimicrobial Stewardship program led by pharmacists and involving multiple disciplines. The program focused on revising the antibiotic formulary and optimizing key points of antibiotic management, using the inpatient Antibiotic Use Density as the core control indicator. This was conducted through three phases: program initiation, implementation, monitoring and control. Clinical pharmacists ensured the long-term operational quality of the program. We evaluated the impact of the program on relevant indicators of antimicrobial consumption in inpatient.

**Results:**

Compared to the pre-implementation year of 2020, the annual Antibiotic Use Density for inpatients across the hospital decreased by 22.28% in 2024, reaching 36.26 defined daily doses/100 patient days. Additionally, the monthly inpatient Antibiotic Use Density in 2024 was significantly reduced (*p* < 0.001), along with the antibiotic usage rate (*p* < 0.05), expenditure on antibiotics per inpatient (*p* < 0.001), and the proportion of antibiotic expenditure relative to total for inpatients (*p* < 0.001). The rational use of antimicrobial agents in inpatient wards has been enhanced. Through targeted management, some antibiotics showed trends of increased or decreased usage. The detection rates of Methicillin-Resistant *Staphylococcus aureus* and Extended-spectrum β-lactamase-producing *Escherichia coli* did not show a significant decrease.

**Conclusion:**

The Pharmacist-Driven Antimicrobial Stewardship program effectively leveraged the managerial roles and professional skills of pharmacists in rational drug use management, resulting in a significant reduction in hospital antibiotic consumption. However, to further validate its effectiveness in reversing bacterial resistance, the program requires longer-term operation and could be considered for regional expansion.

## 1 Introduction

Antimicrobial resistance (AMR) is a major global public health crisis, with the increasing consumption of antibiotics being one of the primary contributing factors (1, 2). The estimated antibiotic consumption increased from 21.1 billion to 34.8 billion defined daily doses (DDDs) between 2000 and 2015 (3), and this number is expected to rise to 75.1 billion DDDs by 2030 (4). Many countries have implemented policies aimed at reducing antibiotic consumption to curb AMR. Over the past decade, China has also responded proactively by introducing several national strategies to optimize antibiotic use. These include the Principles of Clinical Use of Antibiotics (2015 Edition) (5), which serves as a guiding document for clinical use and supervision of antibiotics. Additionally, the National Action Plan for Containing Bacterial Resistance (2016–2020 edition, 2022–2025 edition) has been successively issued. Comprehensive management measures have been adopted at the national level to address bacterial resistance, strengthening supervision across various aspects such as drug research and development, production, distribution, application, and environmental protection.

Antimicrobial stewardship (AMS) has been gaining traction in China for some time, advocating for multidisciplinary team intervention in anti-infective treatment. Several Chinese researchers have reported on their AMS activities, achieving outcomes such as reduced length of hospital stays (LOS), decreased inpatient antibiotic expenditures, and improved rationality in antibiotic use (2, 6–8). However, AMS is a complex system that relies on coordinated efforts across various disciplines. During implementation, issues such as unclear departmental roles, ambiguous task divisions, and vague objectives have arisen, leading to insignificant or unsustainable improvements.

Since 2019, China has introduced the National Tertiary Public Hospital Performance Appraisal commonly known as the “National Examination” for hospitals (9). This term reflects its significance in driving hospital reform, improving healthcare quality, and aligning institutional performance with national health policy goals. The “National Examination” includes inpatient Antibiotic Use Density (AUD) as one of its core indicators, with performance directly impacting hospital rankings. Consequently, inpatient AUD, expressed as defined daily doses (DDDs) per 100 patients-days (DDDs/100 PD) (10, 11), has received greater attention.

Given that the responsibility for monitoring and improving this key indicator is typically assigned to the Department of Pharmacy, pharmacists are well-positioned to lead antimicrobial stewardship efforts at the hospital level. Compared to AMS models that emphasize clinical consultation or point-of-care interventions, a pharmacist-driven approach is considered to integrate technical expertise with administrative oversight, supporting more systematic implementation and policy alignment. This structure has the potential to strengthen accountability and facilitate continuous quality improvement in antimicrobial use.

Deyang People’ s Hospital is a 2059-bed tertiary general hospital located in southwestern China. Prior to 2020, the hospital had persistent difficulties controlling the inpatient AUD. At the beginning of 2020, hospital inpatient AUD reached a peak of 51 DDDs/100 PD in a single month. Therefore, in the dual context of combating AMR and the National Tertiary Public Hospital Performance Appraisal, our hospital initiated a Pharmacist Driven Multidisciplinary Antimicrobial Stewardship Program (PDAMS), with pharmacists as the primary drivers and a top-down design involving multiple departments.

## 2 Materials and methods

### 2.1 Program initiation phase

**FIGURE 1:**
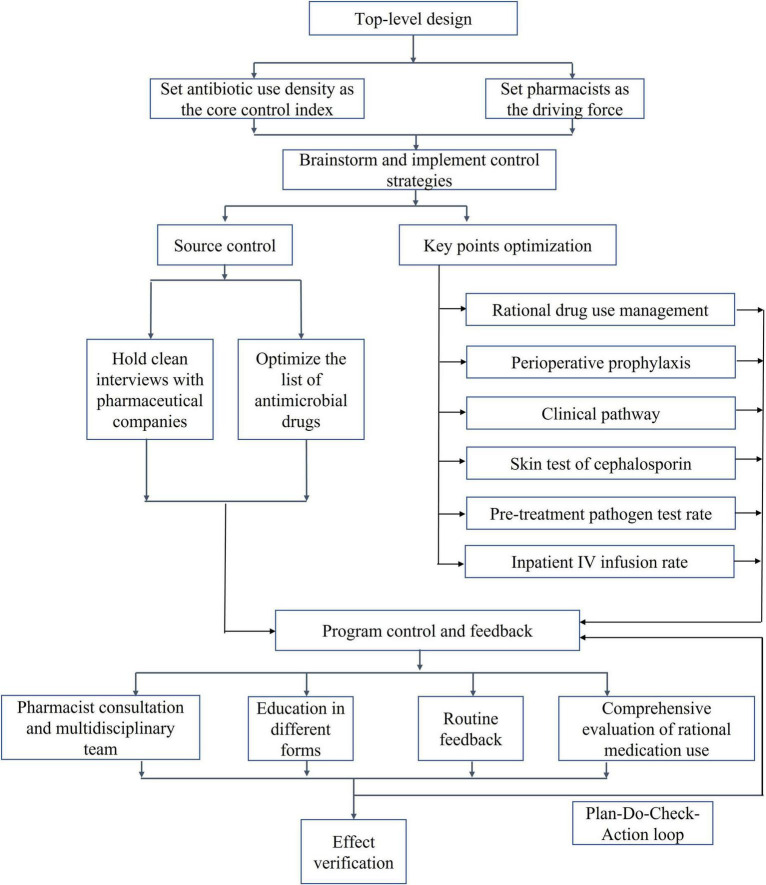
Pharmacist driven antimicrobial stewardship program implementation flowchart.

**FIGURE 2:**
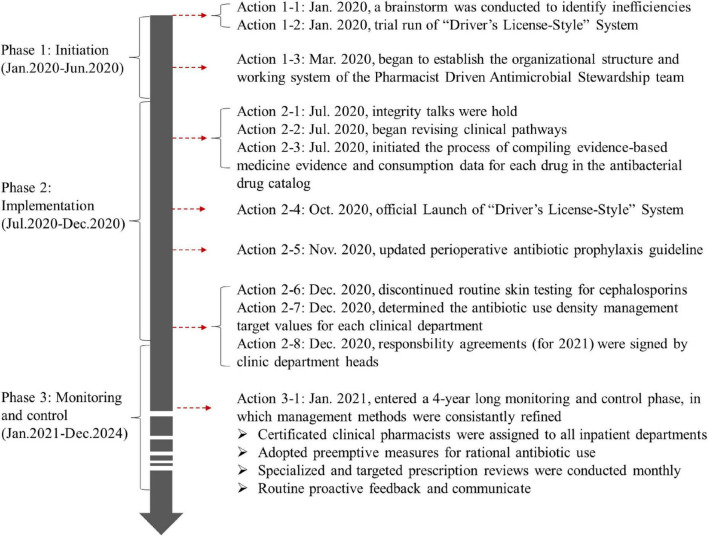
The timeline of the pharmacist driven antimicrobial stewardship program activities.

The first half of 2020 was the initiation phase of PDAMS. The PDAMS team first conducted a brainstorming session to identify the reasons for the previous operational inefficiencies, which mainly included: lack of core control indicators, absence of a driving force, insufficient management of key points, lack of targeted rational drug use control and feedback, and deficiencies in the hospital’s antimicrobial formulary (Figure 1). The program was established as a pharmacist-driven, multidisciplinary antimicrobial stewardship program under the leadership of the hospital director. Referring to national control limit (Table 1), the inpatient AUD was set as the core control indicator, while secondary control indicators included the inpatient antibiotic usage rate and expenditure. The timeline of the PDAMS program activities is shown in Figure 2.

**TABLE 1:** The national control limits of clinical antibiotic use established by the national health commission in 2015.

Antibiotic outcome measures	Control limits
Inpatient antibiotic use density	≤40 DDDs/100 PD
Inpatient antibiotic usage rate	≤60%

The control limits are set for tertiary general hospitals. Inpatient antibiotic usage rate (%) = 100% × Number of hospitalized patients using antibiotics/total number of hospitalized patients in the same period.

The calculation method for inpatient AUD is as follows:


(1) $$\text{DDDs}=\frac{\mathrm{unit}\,\mathrm{strength}\times \mathrm{pack}\,\mathrm{size}}{\text{DDD}}$$

×number⁢of⁢package⁢of⁢antibiotics

(2) average⁢length⁢of⁢stay⁢(LOS)=

$$\frac{\mathrm{total}\,\mathrm{LOS}}{\mathrm{number}\,\mathrm{of}\,\mathrm{inpatient}\,\mathrm{hospitalizations}}$$

(3) Inpatient⁢AUD=

$$\frac{\text{DDDs}}{\mathrm{number}\,\mathrm{of}\,\mathrm{inpatient}\,\mathrm{hospitalizations}\times \text{average}\,\text{LOS}}\times 100$$

Note: Number of inpatient hospitalizations includes hospitalizations for normal deliveries, discharges without delivery, negative diagnostic findings, patients who left without treatment, and healthy individuals discharged following induced abortion or sterilization procedures, among which some cases may have a length of stay less than 24 h (12). Day-case hemodialysis and day chemotherapy services are not included in this definition.

### 2.2 Implementation phase

During the second half of 2020, the program underwent its implementation phase. Our efforts were concentrated on both source control and the management of key points. Source control includes the integrity talks conducted by pharmacists in collaboration with the Hospital Discipline Inspection Committee with manufacturers of antibacterial drugs that rank high in usage and expenditure. Meanwhile pharmacists reviewed and compiled evidence-based medicine (EBM) and consumption statistics for the antimicrobial formulary. The information was then presented to the Pharmacy and Therapeutics Committee for consideration in optimizing the formulary listings.

**FIGURE 3:**
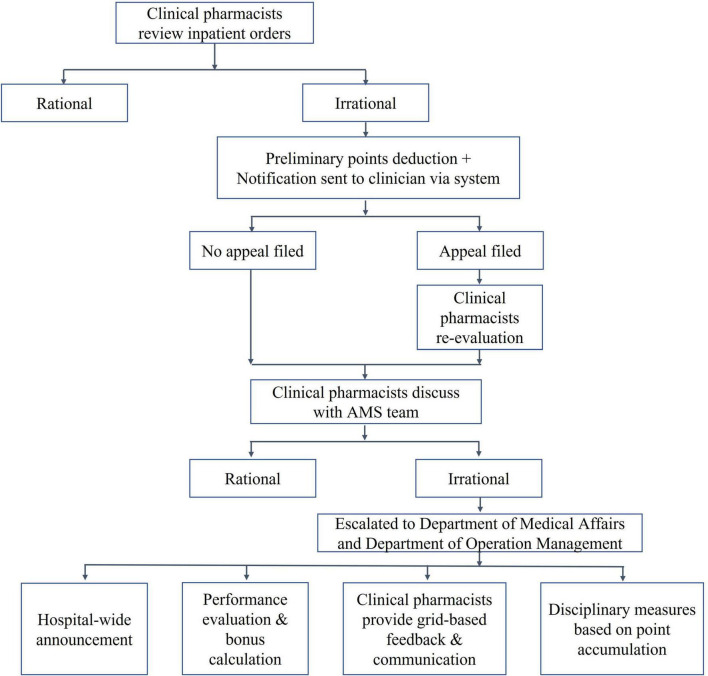
Driver’s-License-Style” System flowchart.

For the optimization of key points, we have initiated a series of multidisciplinary collaborative activities led by pharmacists, including:

Established a “Driver’s License-Style” Point Card System (hereafter, the “Driver’s-License-Style” System) for rational drug use management (Figure 3). The system was first conceived in 2019 and underwent a trial operation phase from January 2020, before being officially launched in October 2020. This initiative was developed based on insights gathered from extensive consultations with clinical departments during its conceptualization stage in 2019, inspired by China’s point deduction method for motor vehicle drivers. The system sets a 6-month period as one scoring cycle, assigning point values ranging from 0.1 to 0.5 to different types of irrational drug use behaviors. Designed primarily as an educational and feedback-oriented tool, the system aims to support clinicians in improving prescribing practices rather than simply imposing penalties. Within each cycle, clinicians who accumulate more than 6 points will be subject to a series of interventions. These include integrity talks conducted by multiple departments, public announcement within the hospital, and a 1-month suspension of prescription rights. Before resuming their duties, they are required to pass an antibiotic knowledge examination. Clinicians accumulating over 12 points face a longer period of suspension and additional training requirements. These measures are intended to foster continuous learning and accountability, and they also influence individual performance evaluations and departmental performance bonuses.

Updated hospital perioperative antimicrobial prophylaxis guidelines that exceed national standards (5). Based on EBM (5, 13–20) and the practical significance of prevention, we further tightened the criteria for prophylactic drug use, including the Preventive indications, timing, dosage and drug selection (Table 2). Additionally, we encourage clinicians to minimize the duration of perioperative antibiotic prophylaxis in accordance with the following principle: a postoperative dose should be avoided if a single preoperative dose is deemed sufficient, and if both pre- and post-operative doses are administered, the duration should not exceed 24 h unless it is clinically necessary.

**TABLE 2:** Typical illustrations of our hospital’s perioperative antibiotic prophylaxis guidelines surpassing national standards.

Category	Detailed description	National standard	Our standard	Supporting evidence
Preventive indications	Risk factor (advanced age)	> 60 years old (5, 20)	>70 years or > 60 years with other risk factors	After discussion by the antimicrobial stewardship team, it was noted that our country has entered an aging society with a large elderly population, and many elderly individuals aged between 60 and 70 are in good health (14, 17).
Preventive indications	Hemorrhoid surgery	No specific guidelines for hemorrhoid surgery. Only recommendations for rectal surgery include cefazolin or cefuroxime ± metronidazole, ceftriaxone ± metronidazole, or cephamycins	Clearly stipulates that antimicrobial prophylaxis is not used for hemorrhoid surgery	The prophylactic use of antibiotics for hemorrhoid surgery remains controversial, with evidence suggesting that prophylactic antibiotics do not reduce the postoperative surgical site infection rate (18).
Timing	Timing of prophylactic antibiotic use before cesarean section	Before skin incision or before cord clamping	Before skin incision	Evidence suggests that the administration of antibiotics before skin incision as a prophylactic measure can reduce the incidence of postpartum endometritis and the overall infection rate, without affecting neonatal outcomes (13, 15).
Dosage	Perioperative prophylactic dosage of cefazolin and cefuroxime	Not explicitly specified	Cefazolin: Initial dose 1-1.5g, followed by 0.5-1g, q8h; Cefuroxime: Initial dose 1.5g, followed by 0.75g, q8h	The purpose of preoperative prophylaxis is to ensure an effective blood drug concentration at the relevant site before skin incision, and only a maintenance dose is required thereafter (19).
Drug selection	Class II incision surgery in urology (entering the urinary tract or via the vagina)	Cefuroxime, cefazolin, or fluoroquinolones (with strict restrictions on the use of fluoroquinolones)	Cefuroxime or cefazolin	The resistance rate to quinolones is high in China (16).

Issued a guideline to discontinue routine cephalosporin skin testing. Since the practice of conducting skin tests for all patients prior to the administration of cephalosporin antibiotics lacks EBM support (21, 22), and there is currently no standardized skin test reagent available for clinical use. The guideline clarify that a skin test is not required before administering cephalosporins, except in cases where there was a documented history of allergy to specific penicillins or cephalosporins.

The Department of Pharmacy, in collaboration with the Department of Hospital Infection Control and the Department of Clinical Laboratory, Medical Affairs department, and Nursing Department, established a multi-disciplinary team. This team conducted various forms of training sessions on microbial specimen submission, including continuing education programs, special topic lectures, and the development of relevant specimen collection and submission protocols. Pharmacists and clinical laboratory technologists established a regular interaction mechanism to jointly confirm challenging microbial test reports. Additionally, pharmacists, in collaboration with hospital infection control physicians, provide quarterly feedback at the hospital level regarding microbial specimen submission.

Access permissions were granted to clinical pharmacists to view clinical pathways, which are structured care plans aimed at ensuring consistent, evidence-based treatment. Clinical pharmacists reviewed and provided feedback on all clinical pathways that included antibiotics. Based on these reviews, the Department of Medical Affairs oversaw the necessary clinical improvements.

Continuous efforts were made to improve the standardized utilization rate of intravenous infusions for inpatients, advocating the principle of “oral administration preferred over intravenous infusion when possible.” For antibiotics, cases where oral administration is feasible but intravenous therapy is used are included in the rational drug use evaluation.

During implementation phase, clinical pharmacists, based on the definitions of various antibiotic-related indicators including inpatient AUD, revised or created relevant statistical reports by presenting criteria to the Information Department. Department of Pharmacy calculated target control values for each clinical department based on their current inpatient AUD status across the hospital and individual clinical departments. These target values were reviewed by the Department of Medical Affairs and verified by the AMS team.

To ensure the project is rigorously implemented, the hospital director emphasized at a mid-level management meeting that “either the inpatient AUD of clinical departments or the positions of department heads must decrease,” and included inpatient AUD targets in the department heads’ responsibility agreements. Since the control of inpatient AUD was directly linked to the positions of department heads, they established AUD evaluation targets for each medical team within their departments. These targets were included in the responsibility agreements, and monthly performance bonuses were assessed at the team level based on these targets.

All policies concerning the rational use of antibiotics were established by the PDAMS team in collaboration with the Pharmacy and Therapeutics Committee, featuring clear and strict reward and punishment measures. Specifically, penalties for inappropriate antibiotic use were set at three times the cost of the misuse for the first occurrence, five times for the second, and 10 times for the third. In parallel, the hospital had established awards such as Outstanding Chief Resident, Devotion Star Award, and top 10 physicians. During the selection process for these awards, deductions under the “Driver’s License-Style” System were taken into consideration. Award winners would receive more opportunities for professional title promotion, position advancement, and external training. Those measures had been thoroughly implemented.

### 2.3 Monitoring and control phase

Following 1 year initiation and implementation period, the program transitioned into a long-term control phase. During this phase, the clinical pharmacist team played a pivotal role. At our hospital, becoming a clinical pharmacist requires at least a master’s degree or an intermediate-level professional title, as well as completion of a 1-year standardized training program for specialized clinical pharmacists organized by the National Health Commission (NHC) (23, 24). Additionally, all clinical pharmacists participating in the PDAMS program had successfully completed the assessment under the “Pei Ying Project” (Pharmacist Training Project in Anti-bacterial/Fungal Infections Diagnosis and Treatment), conducted by the National Institute of Hospital Administration under the NHC (25). During the study, clinical pharmacists hold monthly discussions with the multidisciplinary AMS team on the review of irrational use of antibiotics. They also participated in antibiotic-related academic conferences or seminars on an irregular basis.

Clinical pharmacists were assigned to all inpatient departments across the hospital in a grid-like manner. For example, an anti-infectious clinical pharmacist is required to participate in ward rounds in the Department of Infectious Diseases and also coordinate tasks with other relevant departments, such as General Medicine and Geriatrics. Similarly, a respiratory clinical pharmacist participates in ward rounds in the Respiratory Department while also collaborating with Department of Thoracic Surgery and Department of Emergency Medicine; an ICU clinical pharmacist oversees the ICU and coordinates with the NCU, among others. Following the issuance of each hospital-wide guideline or regulation on antibiotics, comprehensive presentations were delivered to all clinical departments by the clinical pharmacists.

Preemptive measures for rational drug use were implemented, including the establishment of a chief pharmacist on-call system for antimicrobial drugs. This enhanced existing ward rounds and consultations and integrated clinical pharmacists into the multidisciplinary team (MDT) for infectious disease diagnosis and treatment, ensuring their routine participation in antibiotic application decisions. Antibiotics usage was monitored and ranked over defined periods, with monthly limits set for the most frequently used drugs within each clinical unit. Once the limit was reached, further prescriptions required approval through consultation with a clinical pharmacist.

Driven by clinical pharmacists and with full cooperation from the Department of Medical Affairs, stringent measures for rational drug use management were implemented. Regarding the review content, the team continued to conduct previously established specialized reviews, including those for enzyme inhibitor combinations, carbapenems, and the perioperative prophylactic use of antibiotics in Class I/II incision surgeries. Class I incisions are defined as procedures performed under sterile conditions without entry into contaminated or infected areas, thus posing a minimal risk of surgical site infection. In contrast, Class II incisions involve controlled access to anatomical sites that may contain normal indigenous flora but are not considered infected (5, 15). Furthermore, the team conducted targeted reviews monthly on departments and specific antibiotics that had the highest inpatient AUD and antibiotic usage rate. Meanwhile, during the control phase of the PDAMS program, the “

Driver’s License-Style” System was implemented to assist in rational drug use management.

Throughout the program’s operation, the outcomes of rational drug use reviews and pharmaceutical operation metrics for clinical departments were not confined solely to internal network announcements. By the end of each month, clinical pharmacists visited the clinical departments to give feedback and communicate. The content of these sessions included departmental inpatient AUD, antimicrobial drug usage rates, lists of clinicians penalized under the “driver’s license-style” points system, and analyses of irrational drug use. This approach ensured timely feedback and immediate intervention.

The introduction and discontinuation of antimicrobial drugs, the implementation of core measures, and the processes of communication, feedback, and intervention were not isolated events but rather continuous, ongoing efforts. When abnormal fluctuations in control indicators centered on AUD occurred either hospital-wide or within specific departments, pharmacists used the Plan-Do-Check-Action (PDCA) method (26) to integrate the aforementioned reviews, analyses, communications, and feedback, ensuring continuous control.

### 2.4 Data collection

The primary outcome indicator of the program was the inpatient AUD. Secondary outcome indicators included the inpatient antibiotic usage rate, the inpatient expenditure on antibiotics and its proportion of total expenditures, and the DDDs of major antibiotics. These indicators were reported monthly and obtained from the Hospital Information System (HIS). To determine whether changes in inpatient antibiotic expenditure were due to the PDAMS program or the national volume-based procurement (VBP) policy, the impact of the policy was assessed by estimating price differences for antibiotics with the same generic name, dosage form, and strength. These estimated differences were then multiplied by corresponding drug utilization to model hypothetical expenditure in the absence of the VBP. Finally, this amount was added to actual monthly expenditure to derive the Estimated Inpatient Antibiotic Expenditure for 2024. The point deduction data (obtained from a software linked with the HIS) from the “Driver’s License-Style” system was also incorporated into the observation metrics. The detection rate of Methicillin-Resistant *Staphylococcus aureus* (MRSA) was reflected by the resistance rate of *Staphylococcus aureus (S. aureus)* to oxacillin, and the detection rate of Extended-spectrum β-lactamase-producing *Escherichia coli* (ESBL-E) was reflected by the resistance rate of *Escherichia coli (E. coli)* to ceftriaxone.

Data analysis was performed using the Python programming language and the SciPy package. To evaluate the effects of the program implementation, data from 2024 were compared with those from 2020, including inpatient AUD, inpatient antibiotic usage rate, monthly expenditure on antibiotics per inpatient, and the proportion of antibiotic expenditure relative to total for inpatients. The Shapiro-Wilk test was used to assess whether each group of data followed a normal distribution, and the Levene test was applied to determine if the variances between the two groups were equal. Based on the results of normality and homogeneity of variance tests, appropriate statistical tests were selected: if the data were normally distributed and had equal variances, an independent samples *t*-test was used; otherwise, the Mann-Whitney U test was employed. Severe allergy ratio among cephalosporin adverse drug reactions (ADRs) and the differences in antimicrobial resistance between studied years were analyzed using the chi-square test (1). Differences were considered statistically significant when *P* < 0.05.

## 3 Results

The program underwent three phases: 1 year of program initiation and implementation, followed by 4 years of quality control. In terms of inpatient antibiotic consumption metrics, there was a significant decrease in 2024 compared to 2020. The consumption of some antimicrobial agents changed due to the implemented control measures.

### 3.1 Changes in inpatient AUD

**FIGURE 4:**
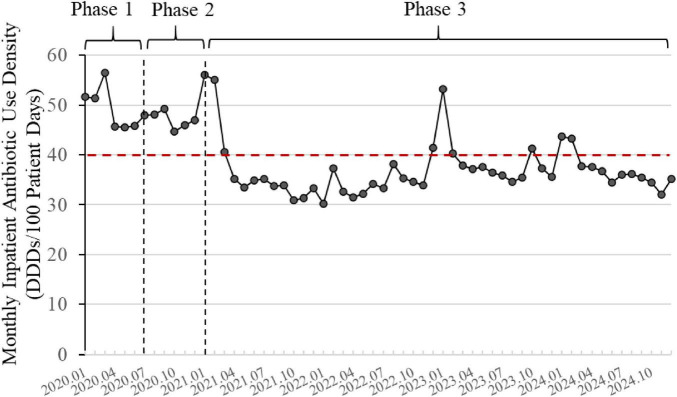
Changes in inpatient antibiotic use density (2020–2024). The line graph is based on monthly data. The red dashed line represents the national control limit for tertiary general hospitals (<40 DDDs/100 patient days). Phase 1: Initiation; Phase 2: Implementation; Phase 3: Monitoring and Control.

Over the 5 years of the program, the changes in inpatient AUD are shown in Figure 4. Overall, there was a downward trend in AUD. The annual inpatient AUD for 2020 was 46.58 DDDs/100 PD, and by 2024, it had decreased to 36.26 DDDs/100 PD, representing a reduction of 22.28%. The monthly inpatient AUD in 2024 was significantly lower than that in 2020 (*p* < 0.001). Compared to 2020, the average annual decrease over the subsequent 4 years was 21.26%.

### 3.2 Changes in inpatient antibiotic usage rate

**FIGURE 5:**
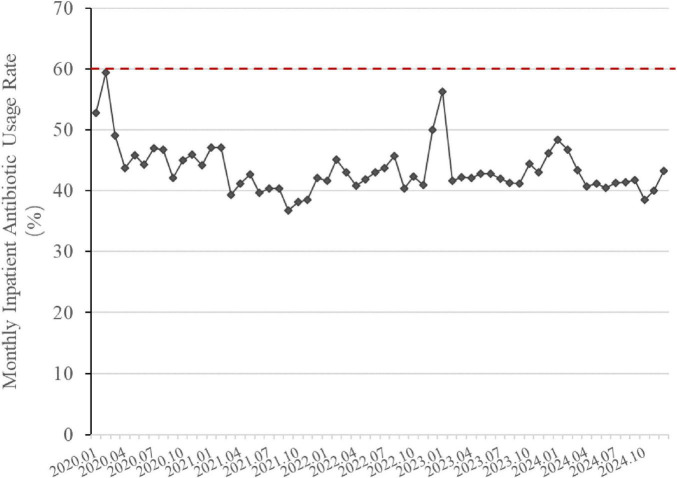
Changes in inpatient antibiotic usage rate (2020–2024). The line graph is based on monthly data. The red dashed line indicates the national control limit for tertiary general hospitals (<60%). Inpatient Antibiotic Usage Rate (%) = 100% × Number of hospitalized patients using antibiotics/Total number of hospitalized patients in the same period.

Figure 5 shows the changes in antibiotic usage rate among inpatients. In early 2020, the inpatient antibiotic usage rates were 52.75% in January and 59% in February. The annual average antibiotic usage rate for 2020 was 46.25%. By 2024, the annual antibiotic usage rate for hospitalized patients had further reduced to 41.57%, marking a decrease of 10.12% from 2020 level. The monthly inpatient antibiotic usage rate in 2024 was significantly lower than that in 2020 (*p* < 0.05). During this timeframe, the inpatient antibiotic usage rate also exhibited a declining trend and remained below the national control limit.

### 3.3 Changes in expenditure and proportion of antibiotics for inpatients

**FIGURE 6:**
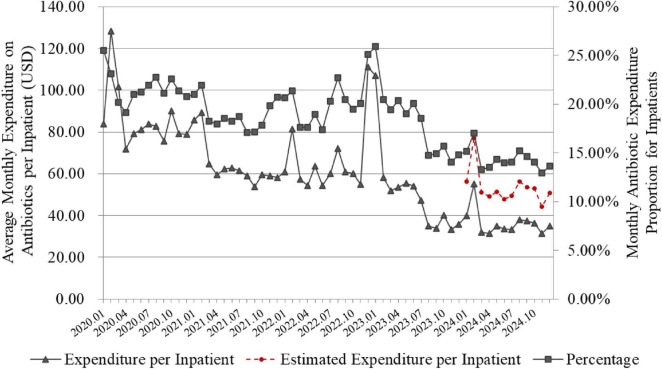
Changes in expenditure and proportion of antibiotics for inpatients. The line graph is based on monthly data. Proportion of antibiotic expenditure in inpatients (%) = 100% × total cost of antibiotics used by inpatients/Total cost of all medications used during the same period. The red dashed line represents the monthly expenditure on antibiotics per inpatient in 2024, after adjusting for the impact of the VBP policy.

The program reduced both the monthly expenditure on antibiotics per inpatients and the antibiotic expenditure proportion (Figure 6). The 2 monthly indicators in 2024 was significantly lower than that in 2020 (*p* < 0.001). In 2020, the average monthly expenditure on antibiotics per inpatient and the antibiotic expenditure proportion for inpatients were $82.77 and 21.65%, respectively. Through project interventions, by 2024, these indicators had decreased to $35.81 per inpatient and 14.27%, respectively. After adjusting for the impact of the VBP on inpatient antibiotic expenditure, the estimated expenditure on antibiotics per inpatient in 2024 was $52.41, which remained significantly lower than that in 2020 (see the red dashed line in Figure 6, *p* < 0.001).

### 3.4 Monthly changes in major antibiotics consumption for inpatients

**FIGURE 7:**
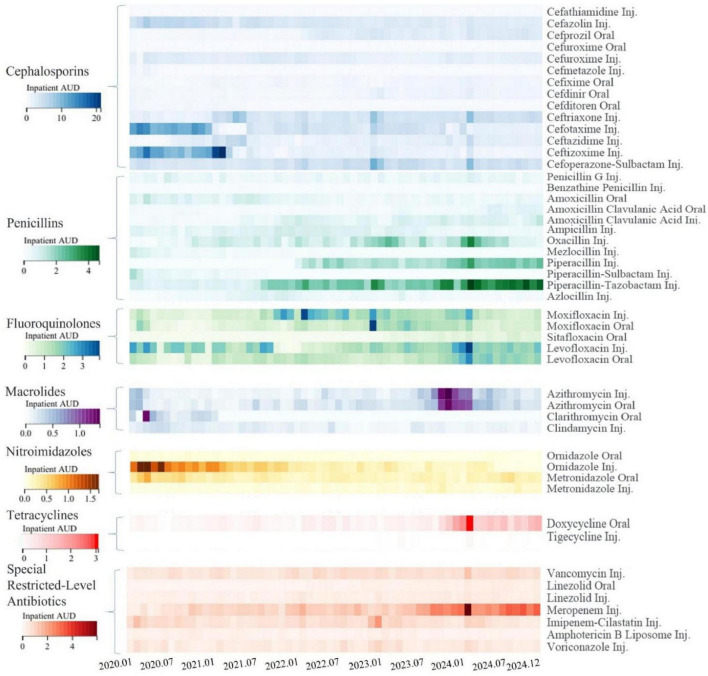
Changes in inpatient antibiotic use density for major antibiotics. The heatmap is based on monthly data, different classes of antibiotics are represented by different color clusters in the heatmap, each with its own scale. Within the same class of antibiotics, the darker the color, the higher the inpatient antibiotic use density (AUD) for that month, indicating greater consumption. “Inj.” denotes injectable formulations, while “Oral” denotes oral formulations.

Over the 5-year implementation of the program, noticeable changes were observed in the consumption of major antimicrobial categories (as measured by inpatient AUD) at our hospital. New drugs were introduced while others were phased out, and usage levels fluctuated with some increasing and others decreasing, as shown in Figure 7. The reduction in antibiotic usage occurred under several circumstances:

Control due to inappropriate use: for example, ceftizoxime, cefotaxime, and ornidazole injections were controlled in 2021, and oxacillin was restricted by the end of 2023.

Tightened usage and treatment duration: for instance, stricter controls were imposed on the indications and treatment duration of cefazolin and cefuroxime for perioperative prophylaxis.

Elimination due to deficiencies and availability of alternatives: Cefathiamidine was phased out due to a lack of EBM support for perioperative prophylaxis, and cefmetazole faced increased restrictions from the National Health Insurance starting in 2020 (limited to severe cases) with its antimicrobial efficacy being replaceable by other agents.

Restriction for Preservation: To preserve the antimicrobial sensitivity of moxifloxacin, starting from late 2021, moxifloxacin was subject to increased control measures, requiring expert consultation prior to use.

Conversely, certain antibiotics saw an increase in usage:

Increased Consumption Due to Control of Similar or Comparable Antimicrobial Agents: For example, the consumption of ceftriaxone and ceftazidime increased starting in 2021, levofloxacin rose from 2022, and with the overall restriction of third-generation cephalosporins in mid-2022, penicillins such as piperacillin, piperacillin-tazobactam, and oxacillin saw increased usage.

Increased Usage Due to Changes in Accessibility: Meropenem entered VBP starting from the end of 2022 and had its National Health Insurance reimbursement restrictions lifted, resulting in higher subsequent usage.

Seasonal Variations or Outbreaks of Infectious Diseases: Winter generally sees higher antibiotic usage, and there was a notable increase in antibiotic consumption during the COVID-19 outbreak peak in late 2022 in China and a mycoplasma pneumonia outbreak peak in late 2023, leading to increased use of azithromycin.

### 3.5 Changes in points deducted by the “Driver’s License-Style” System

**FIGURE 8:**
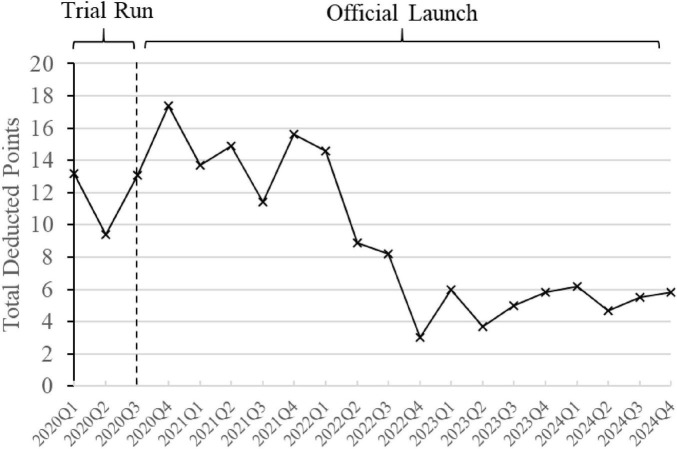
Changes in points deducted by the “Driver’s License-Style” System. The line graph is based on quarterly data, with each data point reflecting the total points deducted for irrational antibiotic use in inpatient wards during that quarter’s review.

Figure 8 reflects the total points deducted quarterly by the “Driver’s License-Style” System, based on the data of irrational antibiotic use in inpatient wards. In the first full calendar year after implementation, 2021, the average points deducted per quarter were 13.9. After continuous interventions, feedback, and communication on rational antibiotic use, the average quarterly deductions in 2024 decreased to 5.55 points, showing a notable reduction.

### 3.6 Change in severe allergy ratio among cephalosporin ADRs

**FIGURE 9:**
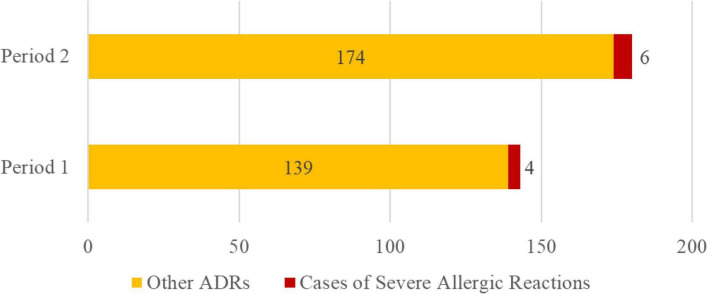
Change in severe allergy cases among cephalosporin ADRs. Note: Period 1: 2018–2020. Period 2: 2021-2024.

The proportion of severe allergic reactions among all cephalosporin ADRs did not significantly increase before and after the discontinuation of routine skin testing (Figure 9). From January 1, 2018 to December 31, 2020 (Period 1), four cases of severe allergic reactions and 139 other ADRs related to cephalosporins were reported. The guideline to discontinue routine cephalosporin skin testing was issued in December 2020. From January 1, 2021 to December 31, 2024 (Period 2), six cases of severe allergic reactions and 174 other ADRs related to cephalosporins were recorded (p > 0.05).

### 3.7 Resistance rates of *S. aureus* to oxacillin and *E. coli* to ceftriaxone

**FIGURE 10:**
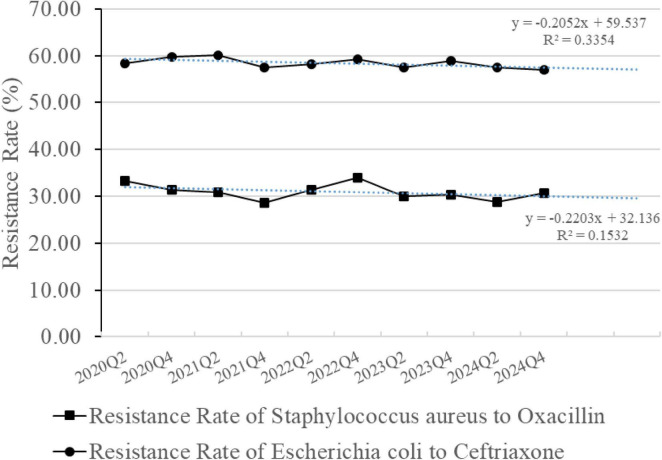
Resistance rates of *Staphylococcus aureus* to oxacillin and *Escherichia coli* to ceftriaxone. The line graph is based on semiannual data. The blue dashed line represents the linear trendline based on the least squares fit.

The hospital-wide bacterial resistance surveillance data were updated semi-annually, specifically at the end of the second quarter (Q2) for the mid-year data and at the end of the fourth quarter (Q4) for the full-year data (Figure 10). Compared with the annual data, the resistance rate of *S. aureus* to oxacillin decreased from 31.40% (243/774) in 2020 to 30.60% (265/866) in 2024 (*p* = 0.728), and the resistance rate of *E. coli* to ceftriaxone decreased from 59.82% (981/1640) in 2020 to 56.90% (1022/1796) in 2024 (*p* = 0.084). However, the downward trend was slow and showed no statistically significant difference.

## 4 Discussion

Inpatient AUD is widely used around the world to reflect the consumption and rational use of antimicrobial agents in healthcare institutions (27). Effectively implementing AMS activities is recognized as a crucial approach to optimizing the use of antibiotics, with pharmacists playing a significant role (7). Wang et al. (8), among the earlier researchers in China to conduct AMS studies, demonstrated through clinical pharmacists’ efforts in setting up antibiotic classification management and intervening in perioperative antibiotic prophylaxis use that there was a decline in AUD and antibiotic usage rates among inpatients over 6 years from 2011 to 2016. Uda et al. (28) reduced the consumption of anti-pseudomonal drugs through pharmacist-led prescription reviews and rational antibiotic use education. Elrefaei et al. (29) deployed anti-infectious clinical pharmacists to implement weekend AMS in ICU and ACU, resulting in decreased Length of Stay. Liu et al. (11) conducted multifaceted AMS before and during the COVID-19 pandemic, effectively reducing the intensity and rate of antimicrobial use among inpatients.

Building upon these foundational studies, our PDAMS model further extends the scope and duration of AMS implementation, incorporating more detailed monitoring indicators such as changes in specific antimicrobial categories, expenditure trends, and targeted quality improvement methods like the “driver’s license-style” antimicrobial management system. Our program integrates national policy requirements, EBM, and real-world hospital operations into a structured, hospital-wide AMS framework driven by pharmacists. Unlike many earlier reports that focus on short-term or department-specific interventions, we emphasize a long-term, systematic approach based on the PDCA cycle, led by hospital leadership and targeting key performance indicators including inpatient AUD and antimicrobial utilization patterns.

Positioning pharmacists at the core of this initiative was of strategic importance. Pharmacists not only provide technical expertise but also undertake administrative responsibilities, such as drafting guidelines for rational drug use and conducting medication practice reviews. Additionally, since inpatient AUD is a core performance indicator in the National Tertiary Public Hospital Performance Appraisal system, its oversight typically falls to the pharmacy department. The operation of PDAMS clarifies roles and tasks in antimicrobial management, enabling proactive refinement and updates of critical aspects according to evolving policies and EBM.

During the operation, the inpatient AUD and usage rates still experienced peaks, primarily occurring during the winter. At the beginning of 2020, the COVID-19 outbreak occurred, but in Deyang, Sichuan, where our hospital is located, there were relatively few positive cases in the community. All positive patients were admitted to our hospital’s isolation ward. The high AUD at that time was due to previous inadequate antibiotic management at our hospital, reflecting only the initial state of our control. The peak at the end of 2022 coincided with another wave of COVID-19 outbreaks in China, which also happened during winter. Therefore, during this period, numerous patients with viral and bacterial co-infections appeared in the community. As a designated medical institution in Deyang City, our hospital took on the primary responsibility for treating these patients.

Short-term fluctuations in antibiotic use rates during periods of local COVID-19 surges were also observed, such as early 2020 and late 2022. These fluctuations likely reflected reduced admissions for non-emergent conditions and changes in clinical practice. Additionally, despite a reduction in surgical volumes (see Supplementary material), we noted an increase in antibiotic use rates among inpatients, which could be attributed to shifts in patient case mix and hospital operations during the pandemic. With fewer elective surgeries, hospitals focused more on managing severe cases, including those requiring intensive antimicrobial therapy. These factors contributed to temporary rises in antibiotic use rates. However, these fluctuations did not compromise the overall effectiveness of our PDAMS, which remained robust throughout the study period. In late 2023, there was a small peak in mycoplasma pneumonia cases, which aligned with the changes in our hospital’s inpatient AUD.

Of course, the peak AUD is not solely the result of an increase in the number of infected patients but is also related to clinical medication habits and even irrational drug use.

Clinical pharmacists analyze the AUD situation across the entire hospital and individual departments monthly, conduct analyses on typical departments and cases, and provide point-to-point communication and feedback to clinicians to intervene together. For example, in early 2020, there was a notable trend among clinicians to prefer ceftizoxime and cefotaxime for the treatment of community-acquired pneumonia (CAP). At that time, the popular dosage of ceftizoxime in the hospital was 3 g every 8 h, resulting in 2.25 DDDs per day. Ceftizoxime and cefotaxime are broad-spectrum cephalosporins primarily active against Gram-negative bacteria. However, common pathogens causing CAP include *Streptococcus pneumoniae* and *Haemophilus influenzae*, with *Mycoplasma pneumoniae* considered in some cases (30, 31). For patients with structural lung disease, *Pseudomonas aeruginosa* should also be taken into account. Therefore, these two antibiotics may not be the optimal choice in all clinical scenarios. After clinical pharmacists educated clinicians on EBM, antibacterial spectra, and the concept of inpatient AUD, the usage of these two cephalosporins gradually decreased, with more targeted selection based on potential pathogens.

In early 2020, there was a trend of using beta-lactam antibiotics combined with ornidazole injections to routinely cover anaerobic bacteria. After discussions, it was determined that most infections did not require routine coverage for anaerobic bacteria, and ornidazole should be used only with definitive evidence. As a result, the usage of ornidazole injections also decreased.

From late 2021 to late 2022, the usage of moxifloxacin was significantly higher. Considering that moxifloxacin is a fourth-generation quinolone with proven activity against respiratory pathogens including Gram-positive bacteria (*Streptococcus pneumoniae*), Gram-negative bacteria (*Haemophilus influenzae*, *Moraxella catarrhalis*), atypical pathogens (*Mycoplasma pneumoniae*, *Chlamydia pneumoniae*), and multidrug-resistant *Streptococcus pneumoniae*, and given its currently low resistance rates, we decided to use it protectively (32). With approval from the AMS team, it was placed under special control, requiring consultation by qualified experts before use. Consequently, the usage of moxifloxacin decreased starting in 2023, while levofloxacin usage increased.

During the mycoplasma pneumonia outbreak peak at the end of 2023, our hospital experienced dual peaks in both intravenous and oral azithromycin use. Clinical pharmacists, aiming to reduce the rate of intravenous infusions and considering the suitability of doxycycline for treating mycoplasma (33), communicated multiple times with departments such as respiratory medicine and pediatrics. As a result, during the mycoplasma pneumonia peak at the end of 2024, the usage of azithromycin injections did not significantly increase, while the usage of oral doxycycline increased.

In the winters of 2023 and 2024, oxacillin DDDs showed peaks, partly due to increased restrictions on broad-spectrum antibiotics. Oxacillin was extensively prescribed for prolonged periods specifically in managing infections like epiglottitis and parapharyngeal space infections. After review by clinical pharmacists and referencing EBM and possible pathogens, recommendations were made to use ceftriaxone or cefuroxime for epiglottitis (Main pathogens: *Haemophilus influenzae, S. aureus, and Streptococcus pneumoniae*.) (34) and amoxicillin-clavulanate, vancomycin, or piperacillin-tazobactam for parapharyngeal space infections depending on whether they were community-acquired or nosocomial, reducing the inappropriate use of oxacillin (35–37).

Among the special restricted level antibiotics, the increase in meropenem usage has been significantly influenced by accessibility and health insurance policies. Since December 2022, meropenem entered the VBP list, resulting in a substantial price reduction. Additionally, starting in 2023, the Chinese National Health Insurance Directory removed the restriction of “limited to severe infections caused by multidrug-resistant organisms” for meropenem. This change led clinicians and patients to prefer meropenem more frequently for severe infections. As a broad-spectrum carbapenem antibiotic, meropenem remains an essential option for treating life-threatening bacterial infections according to clinical guidelines. Nevertheless, this expanded access underscores the necessity for stricter indication review and continuous stewardship oversight to ensure its appropriate and judicious use.

The implementation of “Driver’s License-Style” System was not without challenges. In 2021, we encountered significant resistance from clinicians, and the level of point deductions remained relatively high, reflecting ongoing issues with irrational antibiotic use. Clinical pharmacists did a great deal of work during control and monitoring phase. By collecting EBM data and summarizing issues related to irrational antibiotic use, they provided monthly feedback and engaged in repeated proactive communication. This effort led to a shift among clinicians, who went from passively accepting the “Driver’s License-Style” System for rational drug use to actively endorsing this control approach. Across the hospital, similar irrational practices related to antimicrobial agents have been observed. However, for specific clinicians, after receiving acknowledged feedback and communication, few intentionally repeat such errors.

In the course of program control, there were instances where clinical pharmacists and doctors could not reach an agreement on certain challenging issues related to antimicrobial use. In such cases, clinical pharmacists would initiate a multidisciplinary discussion involving experts from medical affairs, infection control, microbiology labs, infectious diseases departments, internal medicine, surgery, and other relevant departments to resolve the issue and form a hospital-wide consensus. For example, for inguinal hernia surgeries with mesh implants, clinical pharmacists believed that these were clean Class I incision surgeries and should not require routine prophylactic anti-microbial use in principle (5, 38). However, clinicians argued that the presence of an implant constituted a high-risk factor for infection, necessitating prophylactic use. After a multidisciplinary discussion, it was agreed that these procedures are relatively simple, in-volve clean sites with minimal surgical areas, and utilize sterile implants, concluding that prophylactic use was unnecessary. Subsequently, clinicians agreed to follow the multidisciplinary discussion’s recommendations, focusing more on surgical technique, disinfection, and hand hygiene, and refraining from routine prophylaxis (except in cases involving additional high-risk factors such as diabetes or immunosuppression).

The changes in the expenditure on inpatient antibiotics are influenced by both usage variations and VBP policies. During several winter peaks in usage, the expenditure on antimicrobial agents also rose sharply. Additionally, the national VBP aims to ensure drug quality and supply while reducing the financial burden of medications for related diseases (39). The PDAMS program promotes the implementation of centrally procured drugs within the hospital and strengthens the clinical monitoring and management of antimicrobial use. We strictly avoid situations where doctors use antibiotics without indication, overuse them, make inappropriate drug selections, or use improper combination therapy to meet the assigned procurement targets for their departments, in order to prevent adverse impacts on patient treatment.

The national VBP policy has had a significant impact on reducing antibiotic expenditure, but it is not the sole factor. Firstly, not all antibiotic varieties were included in the VBP program; secondly, even for those included, according to the “one drug with two dosage forms/strengths” policy, we can still retain a non-VBP variety. Moreover, the VBP policy affects not only antibiotics but also other drugs. As shown in Figure 6, besides the reduction in antibiotic expenditure, the proportion of antibiotic expenditure relative to total drug expenditure has also decreased. Due to variations in the contracted antibiotic products and their prices allocated to our hospital across different periods and batches, we analyzed the portion of antibiotics included in the VBP program within our hospital’s 2024 antibiotic list. By estimating and incorporating the total expenditure changes due to VBP price adjustments, the per capita antibiotic expenditure in 2024 remained significantly lower than in 2020. This indicates that the implementation of the PDAMS project has played an active role in reducing inpatient antibiotic expenditure.

Discontinuing routine skin testing for cephalosporins represents an important shift in clinical practice, aimed at reducing unnecessary medical interventions and resource waste while ensuring patient safety. Our hospital began exploring the feasibility of eliminating routine cephalosporin skin testing prior to the release of the Chinese National Health Commission’s guideline *Skin Testing for β-Lactam Antibiotics* in April 2021 (40), and shifted the focus of our work toward early monitoring and intervention for severe allergic reactions. This effort provided real-world data to support this policy change. By comparing the incidence of severe allergic reactions and other adverse drug reactions before and after the discontinuation of skin testing, no significant increase in the proportion of severe allergic reactions was observed. These findings are consistent with previous studies (21, 22) and further validate the scientific basis and safety of this policy adjustment.

The program adopted several quality control methods, such as the “Driver’s License-Style” System, grid-based alignment, and PDCA cycles, which contributed significantly. The innovation of the “Driver’s-License-Style” System lies in the fact that rewards and penalties are not only reflected in doctors’ performance but also in their professional reputation. Monthly deduction details are posted on the internal network, and prescription rights are suspended when points exceed the limit, effectively drawing doctors’ attention. Grid-based alignment of clinical pharmacists with clinical departments not only disseminates the latest pharmaceutical information and rational drug use practices but also ensures comprehensive handling of clinical feedback. Assigning specialized clinical pharmacists to these roles allows for more professional problem-solving. The integration of the “Driver’s-License-Style” System with the grid-based pharmacist alignment created a synergistic effect, in which performance accountability was reinforced through visible incentives and penalties, while clinical pharmacists provided frontline support to ensure appropriate antimicrobial use. PDCA, as an advanced management model, effectively enhances management quality. Through the PDCA cycle, the program creates a virtuous loop in antimicrobial drug usage management, enhancing healthcare providers’ sense of responsibility while achieving effective control of inpatient AUD.

The PDAMS project achieved certain successes in its first 5 years, but there are still some limitations. The detection rates of MRSA and ESBL-E in our hospital, as reflected by the resistance rates of *S. aureus* to oxacillin and *E. coli* to ceftriaxone, respectively, did not show a significant decrease within the study period, which is consistent with the findings of other studies (28, 41). The diversity of patient origins, combined with broader factors such as regional prescribing patterns and patient referral networks, makes it challenging to observe rapid declines in resistance rates despite reductions in overall antibiotic use. In the coming years, we will further advance rational antibiotic use through embedded clinical pharmacists and enhanced decision-support systems, while expanding PDAMS across Deyang City and its surrounding areas to promote standardized and homogenized antimicrobial stewardship practices and strengthen coordinated pharmaceutical quality control.

## 5 Conclusion

During this 5-year program, we successfully reduced key antimicrobial consumption indicators, including inpatient AUD, antibiotic usage rate, and expenditure, through the implementation of PDAMS. This structured, pharmacist-driven model enabled sustained improvements in rational antibiotic use at the institutional level, distinguishing it from earlier localized or short-term interventions reported in China. We also optimized the antimicrobial formulary and enhanced prescribing practices across inpatient wards. Although no significant changes were observed in MRSA or ESBL-E detection rates, PDAMS has established a sustainable framework for continuous quality improvement through the PDCA cycle, ensuring ongoing optimization of antimicrobial use. Looking ahead, we aim to expand our model regionally to promote collaborative antimicrobial management and help curb antimicrobial resistance.

## Data Availability

The original contributions presented in this study are included in this article/Supplementary material, further inquiries can be directed to the corresponding authors.
